# In-Vehicle Communication Cyber Security: Challenges and Solutions

**DOI:** 10.3390/s22176679

**Published:** 2022-09-03

**Authors:** Rajkumar Singh Rathore, Chaminda Hewage, Omprakash Kaiwartya, Jaime Lloret

**Affiliations:** 1 Department of Computer Science, Cardiff School of Technologies, Cardiff Metropolitan University, Cardiff Llandaff Campus, Cardiff CF5 2YB, UK; 2 Department of Computer Science, Nottingham Trent University, Clifton Campus, Nottingham NG11 8NS, UK; 3 Department of Communications, Universitat Politècnica de València, 46022 Valencia, Spain

**Keywords:** machine learning, cryptography, cyber attacks, cyber security, intrusion detection system, smart intelligent vehicles, in-vehicle network, controller area network (CAN)

## Abstract

In-vehicle communication has become an integral part of today’s driving environment considering the growing add-ons of sensor-centric communication and computing devices inside a vehicle for a range of purposes including vehicle monitoring, physical wiring reduction, and driving efficiency. However, related literature on cyber security for in-vehicle communication systems is still lacking potential dedicated solutions for in-vehicle cyber risks. Existing solutions are mainly relying on protocol-specific security techniques and lacking an overall security framework for in-vehicle communication. In this context, this paper critically explores the literature on cyber security for in-vehicle communication focusing on technical architecture, methodologies, challenges, and possible solutions. In-vehicle communication network architecture is presented considering key components, interfaces, and related technologies. The protocols for in-vehicle communication have been classified based on their characteristics, and usage type. Security solutions for in-vehicle communication have been critically reviewed considering machine learning, cryptography, and port-centric techniques. A multi-layer secure framework is also developed as a protocol and use case-independent in-vehicle communication solution. Finally, open challenges and future dimensions of research for in-vehicle communication cyber security are highlighted as observations and recommendations.

## 1. Introduction

**Figure 1:**
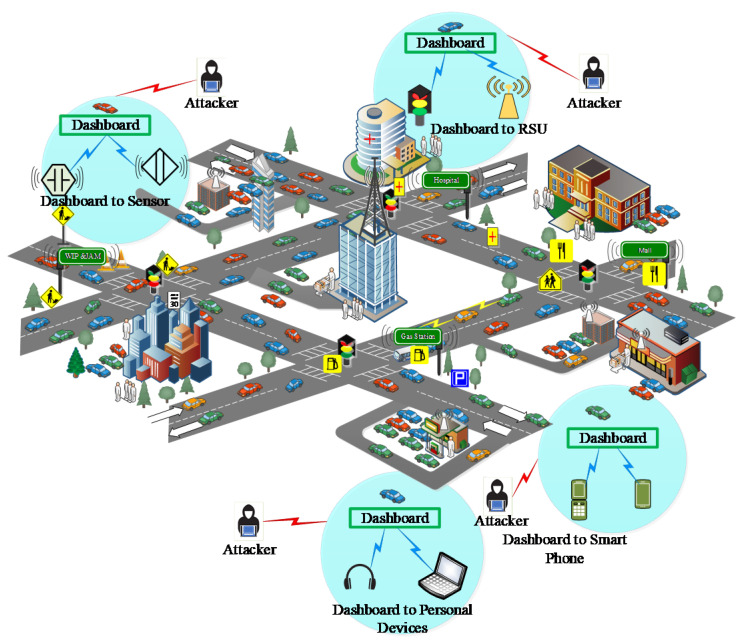
In-Vehicle security scenarios with possible threats.

The current era is the witness of tremendous development in in-vehicle automotive technologies since modern intelligent vehicles can be considered cyber-physical systems with excellent capabilities to connect with external infrastructures [1]. The in-vehicle technology enabled modern intelligent vehicles should not be perceived similar to mechanical systems, but the integrated architecture consists of million lines of complex code to provide various real-time information to vehicle occupants. Current advancements in in-vehicle communication technologies enable the more refined in-vehicle dashboard centric communications including with smart phone, sensors, earphone or laptop, and roadside units (see Figure 1). The embedded hardware enabled in-vehicle short range communications need urgent attentions considering cyber security risks [2]. Recently, we can see a high inclination in research trends towards secure in-vehicle network architectures. Researchers are developing new protocols, and the development of new smart applications are the outcome of this growth [3]. There is an urgent need for the development of efficient protocols for automotive industries which should be fully compatible with current trends and technologies [4]. Next, Figure 1 illustrates the in-vehicle communication security scenarios with security threats.

The advanced in-vehicle functionality in modern intelligent vehicles is provided by electronic control units (ECUs) equipped with the vehicles. Further, these units are connected via serial buses. Controller Area Network (CAN) protocol is responsible for maintaining effective in-vehicle communication among these modules in modern intelligent vehicles [5]. It is a fact that modern in-vehicle technology enabled intelligent vehicles with smart connectivity and computerization have various unique features such as enhanced road safety, connectivity with the outside world, many new services for enhancing the customer experience, etc. However, on the other aspects, these connected smart features have paved the way for hackers to hijack vehicles functionalities, since these features are exposing the vehicle to possible attacks. Hackers may open a new attack surface by accessing the electronic in-vehicle components of modern intelligent vehicles [6].

The existing protocols of the in-vehicle network have various vulnerabilities, such as ID-based arbitration mechanism for contention resolution, unavailability of message authentication and encryption, etc. [7]. There is an urgent requirement for the security of modern intelligent vehicles since adversaries may use existing vulnerabilities and attack the modern intelligent vehicle which may lead to hazards to life and damage to the vehicle on the road. Recent decades are witness of advancements in technology for modern smart intelligent vehicles as well as self-driving vehicles. The modern automotive industries have seen several breakthroughs due to enhanced connectivity, and significant development towards communication channels and access points. On the other hand, these developments open the door for new challenges consisting of security and privacy of data [8]. The safety of life is at the stake due to these cyber security vulnerabilities. Since modern connected vehicles are growing with efficient communication capabilities and sharing critical safety-related information with nearby vehicles as well as with infrastructure around in real-time [9]. This growth is responsible for dynamic and rapid change in the automotive cyber security environment. The cyber security issues should be handled with utmost priority with the detection of any vulnerabilities at the earliest possible time. Cyber security issues may jeopardize modern intelligent vehicles on roads [10,11].

Artificial Intelligence (AI) and Machine Learning (ML) techniques have been used for solving a variety of in-vehicle domain issues [12]. Currently, these techniques have transpired as a reasonable solution for complex application domains. One of the complex application domains is the security of in-vehicle networks [13]. Recently, research innovations have shown that ML-based schemes can effectively address security issues in in-vehicle networks [14]. Since the modern intelligent vehicle network consists of wireless communication between vehicles or infrastructure and increased connectivity has widened the security holes thereby making it vulnerable to a large number of different types of attacks [15]. Additionally, various researchers have significantly contributed in this direction by utilizing machine learning techniques and their variants for developing security frameworks to identify potential attacks and guard against several security-related issues in modern vehicular networks [16]. In the past, cryptographic approaches have been used to propose solutions to handle various types of security issues. For authentication in in-vehicle networks, some traditional authentication techniques include biometric security techniques, key-based authentication, and password protection, etc. The main limitation of cryptographic approaches in handling security issues of in-vehicle networks is low accuracy in validating whether the transferred value is real or spoofed. Additionally, cryptographic approaches are generally implemented in low-powered vehicle security systems [17].

In this context, this paper presents a critical survey of in-vehicle communication focusing on following research questions:

- What are the main components of in-vehicle network?
- Why there is a need to secure in-vehicle network environment?
- Which are the main approaches for securing the in-vehicle network?
- What kind of challenges exist in securing the in-vehicle network?
- Where the future research on in-vehicle cybernetics will move forward?

It first identifies the broad categories of attacks on in-vehicular system. These attacks are classified into four major categories as sensor initiated, infotainment initiated, telematics initiated, and direct interface initiated. Then, we investigate and summarize the available defenses against these in-vehicle cyber-attacks and classify them into two major categories including machine learning-based approaches and cryptography-based approaches. We provide detailed survey and comparison of cryptographic approaches as well as machine learning approaches for designing security solutions against several cyber threats on in-vehicular system. We have developed multi-layered in-vehicle security framework. Open research challenges and future directions for preventing attacks on in-vehicle network systems have been discussed. We are sure that this systematic survey provides the foundation stone about in-vehicle networks’ attacks and security solutions based on machine leaning as well as cryptography techniques and it is therefore will play a crucial role in serving as a groundwork and point of reference for variety of stakeholders with different levels. The contribution can be summarized as below:

1. In-vehicle communication network architecture is presented considering key components, interfaces, and related technologies.
2. The protocols for in-vehicle communication have been classified based on their characteristics, and usage type.
3. Security solutions for in-vehicle communication have been critically reviewed considering machine learning, cryptography, and port centric techniques.
4. A multi-layer secure framework is also developed as a protocol and use case independent in-vehicle communication solution.
5. Finally, open challenges, and future dimension of research for in-vehicle communication cyber security is highlighted as observation and recommendation.

The rest of the paper is organized in following sections. Section 2 presents the architecture and components of in-vehicular communication system. Section 3 briefly describes the classification and characteristics of in-vehicle automotive protocols. Section 4 illustrates the broad classification of attacks on in-vehicle communication system. A comprehensive survey of security solutions for in-vehicle network against malicious threats is presented in Section 5. A multi-layered in-vehicle security framework is proposed in Section 6. Open challenges and future research directions are highlighted in Section 7, followed by conclusions in Section 8.

## 2. Architecture and Components of In-Vehicular System

In-vehicle networks (IVNs) are an emerging research field in modern vehicular networks. The in-vehicle network architecture consists of several core components, namely Sensor Domain consisting of high precision sensors, Chassis Domain, Infotainment Domain, Telematics Domain, Powertrain Domain, etc. For effective communication between these core components of in-vehicle networks, protocols play a major role such as Ethernet, FlexRay, and Controller Area Network (CAN), etc. The rapid growth in connectivity among transportation facilities integrated with modern advanced technologies, for example, V2X-communications, have resulted in widening the security holes and subsequently, attackers can get access to the in-vehicle network.

### 2.1. Internal Configuration of Electronic Control Units

**Figure 2:**
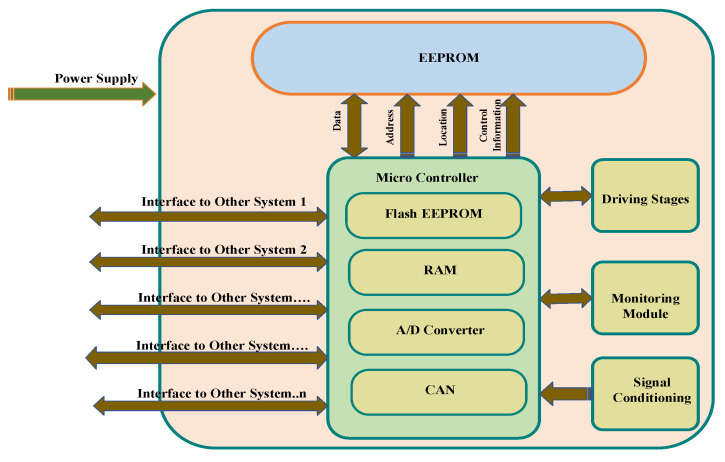
Internal Configuration of ECUs.

Critical information is exchanged among different electronic control units (ECUs) installed in the vehicle. In advanced modern vehicles, as the number of ECUs increases, the complexity level in in-vehicle networks also increases since each distinct component are having different requirements in terms of bandwidth and latency. The number of ECUs is continuously rising in modern intelligent vehicles to enable the modern advanced vehicle with a variety of new functionality for safety, security, convenience, etc. Additionally, for the interconnections of a large number of ECUs, several in-vehicle protocols have been developed and research is still going on with more advanced features. Additionally, ECUs are usually connected with more than one bus network due to their diversified functionality including controlling and monitoring the vehicle [18]. Figure 2 demonstrates the internal configuration of ECU in detail.

### 2.2. In-Vehicle Network Architecture 

**Figure 3:**
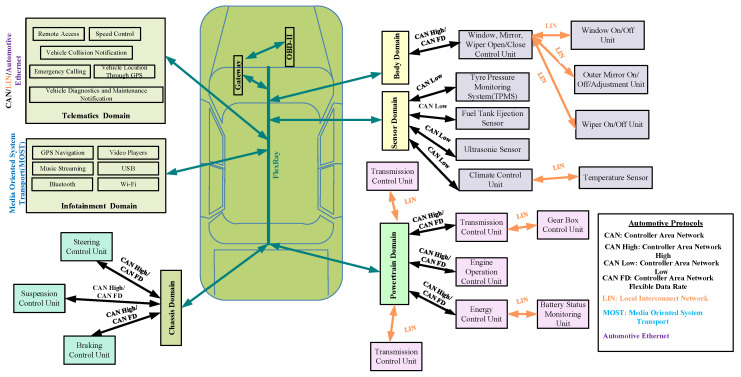
In-Vehicle Network Architecture with Automotive Protocols.

In-vehicle networks which are also known as internal communication networks are responsible for interconnecting various components inside modern intelligent vehicles. ECUs, gateways, sensors, actuators, etc. are considered the main core components inside modern intelligent vehicles. Additionally, the modern intelligent vehicle has various units, namely Telematics Domain, Infotainment Domain, Chassis Domain, Powertrain Domain, Body Domain and Sensor Domain, etc. Sensors give inputs to these electronic units for further computation [19]. Figure 3 presents the general architecture of in-vehicle network.

It is a fact that tiny sensors are the backbone of modern intelligent vehicles since sensors typically recognize and immediately report the problem for resolution example including service due, component fault, etc. Sensors observe various key events such as car speed, fuel, temperature, speed of crankshaft’s rotation, tire pressure, oxygen ratio in the exhaust gases, air density in the engine, etc. therefore tiny sensors should have high standards, such as precision, resolution, sensitivity, accuracy, low power consumption with less noise. The observation and reporting of these events help in the early detection of the problem and result in the prevention of vehicle damage. Autonomous vehicles have sensors of different types ranging from electric, mechanical, optical, sound, image, and light sensors, etc. 

### 2.3. Classification of In-Vehicle Network Architecture

**Figure 4:**
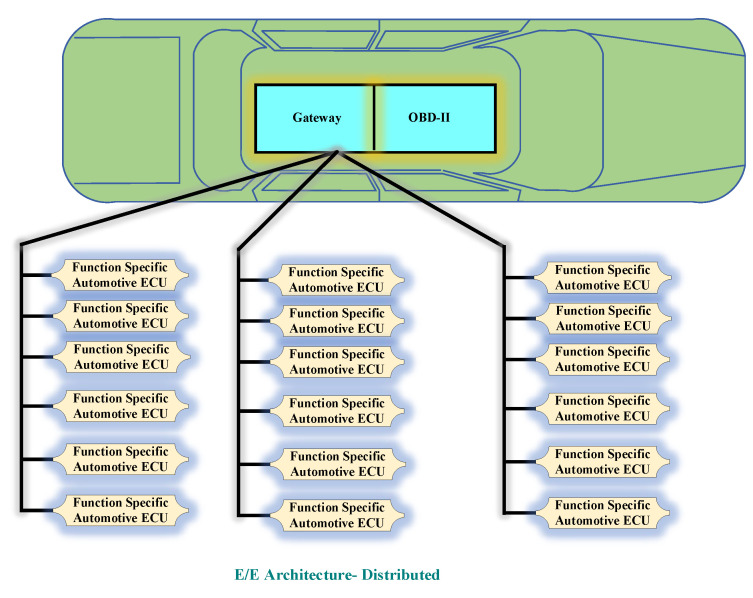
E/E Architecture-Distributed.

The in-vehicle network architecture can be classified into three types. The first classification consists of the central gateway and this type of architecture is known as distributed electrical and electronics(E/E) type. In the second classification, multiple operational domains are connected via a central gateway and this type of architecture is known as the domain-centralized electricals and electronics(E/E) type. The third type of in-vehicle network architecture is known as future E/E architecture or zonal architecture. This architecture has a centralized high-performance computing unit (HPCU) which is useful in reducing the complexity of previously existing two architectures [20]. Figure 4 illustrates the distributed electrical and electronics (E/E) architecture.

The two main components of this architecture are function-specific ECUs along with central gateway. The controller area network (CAN) bus is used for the interconnection. A strong collaboration among the ECUs can be achieved with the help of the central gateway. Therefore, complex functions, such as cross-functional connections as well as adaptive cruise control, can be executed efficiently in this type of architecture with the help of a centralized gateway.

The main limitation of electrical and electronics(E/E) type in-vehicle network architecture is increased communication overhead since different ECUs communicate via a central gateway. For addressing this limitation, an architecture was developed based on several functional domains for in-vehicle network architecture in which different functional/operational domains are interconnected via the central gateway. Additionally, the communication load on the central gateway is significantly reduced since the majority of communication happens within these operational/functional domains themselves. This architecture has scalable capability because more functional domains can be added easily [21].

**Figure 5:**
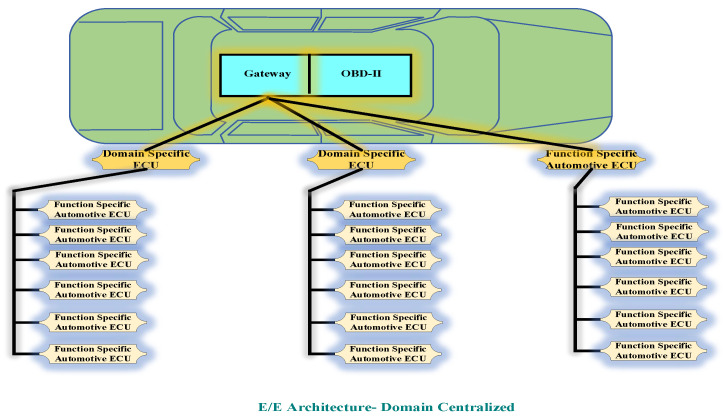
E/E Architecture-Domain Centralized.

In this architecture also, the central gateway plays a crucial role. The main feature of this type of architecture is the use of function-specific automotive ECUs and domain-specific ECUs. Additionally, through CAN bus and Ethernet connections, function-specific automotive ECUs are connected to domain-specific ECUs. This architecture is much more efficient than the previous architecture in terms of handling stringent complex functions. With the help of the consolidation of functions. Domain centralized architecture has become extremely elaborate over time. The autonomous driving feature requires a large number of sensors and actuators which results in higher data processing and bandwidth requirements, and subsequently, the architecture becomes more complex in these scenarios. Figure 5 depicts the domain centralized E/E architecture [22].

**Figure 6:**
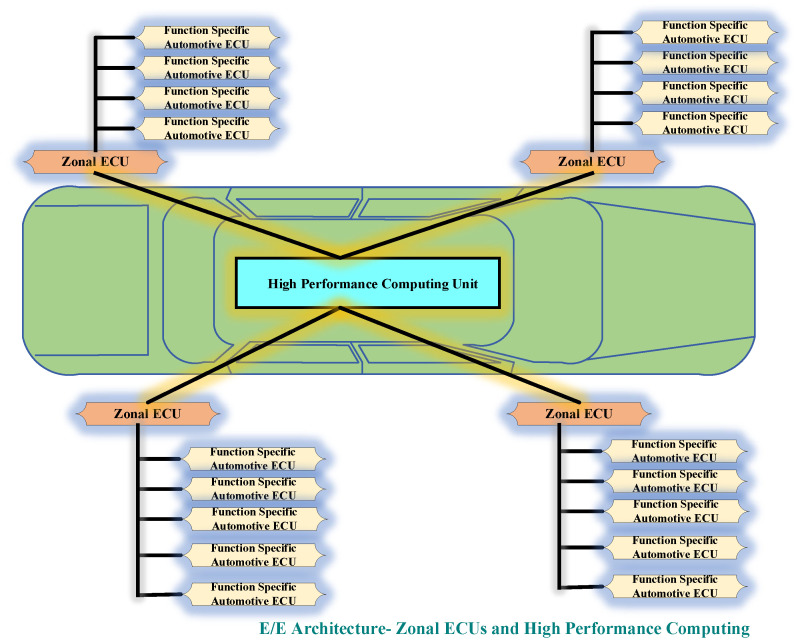
E/E Architecture-Zonal ECU and High-Performance Computing.

The third type of in-vehicle architecture is futuristic architecture also known as zonal architecture. The architecture reflects futuristic smart vehicle functions as well as technologies with a significant reduction in weight and cost. The three main components of the zonal architecture are function-specific automotive ECUs, HPCU, and zonal ECUs. In this architecture, the central controller is HPCU which is responsible for processing all data received from several different zones of the vehicle. The data is transferred from one zone to another through HPCU which works as a central gateway. To match the high bandwidth and speed requirements for data transmission in the vehicle network, the Ethernet connections are utilized for the connection of the ECUs and HPCU. The unique feature of this futuristic architecture is support for the virtual domain. For HPCU, the two key functionalities are transferring the embedded functions into the cloud and the next feature provides software update/download support over the air (OTA) [23,24]. Figure 6 presents the zonal architecture.

The door is open for significant opportunities in the automotive sector by transferring consumer electronics technology and information technology to the automotive sector. The only factor in this transformation is a lot of adaptions. In the current era, rapid changes can be seen in the automotive electronics architecture [25].

## 3. Classifications and Characteristics of In-Vehicle Automotive Protocols

In advanced vehicles (electric, hybrid, driverless), stringent real-time information is exchanged among different modules for smooth function of the vehicle and this requirement is fulfilled by the rapid development of various applications of CAN. Other heterogeneous and complex architectures of in-vehicle networks include networks such as Media-Oriented Systems Transport (MOST), FlexRay, Local Interconnect Networks (LIN), and Automotive Ethernet (AE). Table 1 describes the classification of protocols for in-vehicle network communications.

### 3.1. Controller Area Network (CAN)

For in-vehicle communications, the CAN protocol is generally mainly used. CAN packets are transmitted between multiple ECUs through inter-connected buses. The broadcast communications mechanism is used in the CAN protocol. The CAN protocol has various advantages such as simplicity, low network complexity, and reduced wiring costs since CAN utilizes the multiplex wire architecture for eliminating the need of complex excessive wiring for communication among different ECUs. CAN is unable to provide real-time performance which is a crucial factor for applications related to critical security. the CAN protocol is not able to handle security challenges. The lack of authentication mechanism and encryption is considered the main limitation of the CAN protocol. Additionally, the rapid advancement in automotive applications require support from high bandwidth backend protocols but CAN has bandwidth limitations [26].

### 3.2. Local Interconnect Network (LIN)

LIN is a single wire network for connecting sensors and actuators. Reliability of LIN is not up to the mark as compared with CAN thereby it is not suitable for time critical applications. LIN utilizes the parity bits and checksums for detecting the incorrect messages in the network [27].

### 3.3. FlexRay Protocol

FlexRay protocol utilizes two parallel channels for data transmission in synchronous as well as asynchronous mode. FlexRay can be utilized for time critical applications. Although FlexRay has reliability and fault tolerance features, but implementation cost is very high. FlexRay handles logical errors by using checksums and redundancy mechanisms [28].

### 3.4. Media-Oriented Systems Transport (MOST)

The MOST protocol has been developed by domestic digital bus. MOST protocol support synchronous as well as asynchronous mode for data transmission. MOST protocol also support the GPS applications and radio. Although MOST protocols satisfy the infotainment requirements, but MOST protocols failed to provide bandwidth requirements when the requirement is increased exponentially [29].

### 3.5. Automotive Ethernet

This protocol is considered as physical layer standard in the automotive domain. Due to the diversified capability of this protocol, it can be used in more advanced applications in vehicles such as advanced driving assistance systems, etc. The main advantage of this protocol is the reduced wiring cost since it supports switched network technology [30].

## 4. Classification of Attacks on In-Vehicle Network System for Possible Entry Points

**Figure 7:**
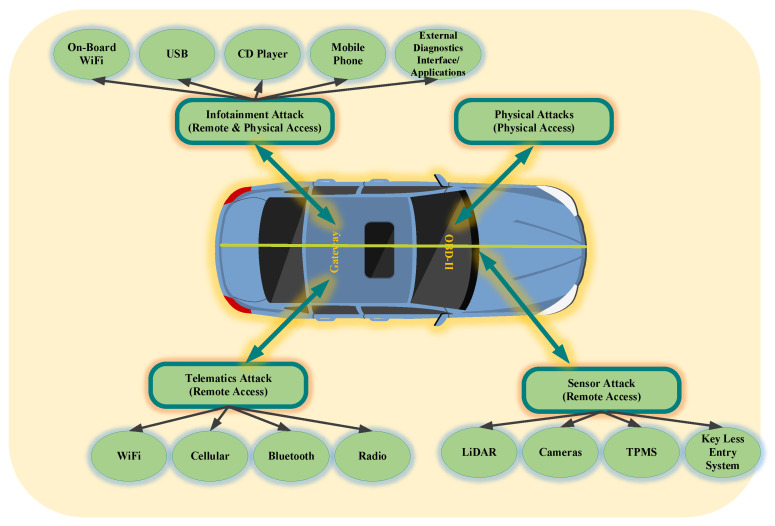
Classification of in-vehicle network attacks.

This paper first identifies the broad categories of attacks on in-vehicular system. These attacks are classified into four major categories as sensor initiated, infotainment initiated, telematics initiated, and direct interface initiated as shown in Figure 7. There are generally two attack vectors, namely wireless access and physical access, attackers are using these attack vectors to get access to the internal networks of the vehicle. External inputs use these interfaces so that ECUs can be exploited. The attackers may use software bugs, the vehicle’s remote key (via the internet) and much more to exploit the ECUs easily. The in-vehicle network has several security issues, research is going on to develop an advanced security framework. Wireless networks can be used for exploiting the bus system of the in-vehicle network.

### 4.1. Entry points to Smart Vehicles

**Figure 8:**
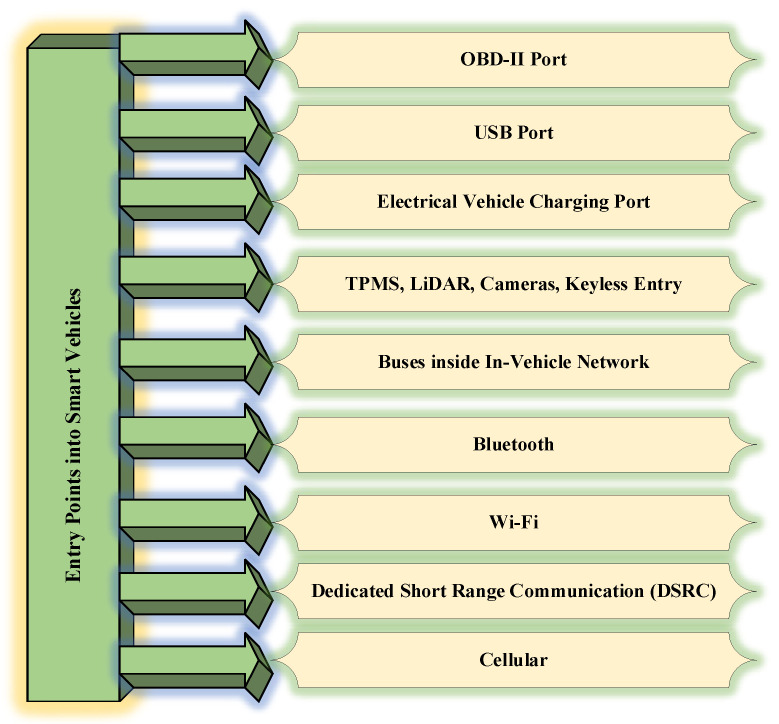
Illustrating entry points to smart intelligent vehicles.

Smart intelligent vehicles have lots of features due to rapid development in automotive technologies. No security mechanism is sufficient enough to handle all security threats. With tremendous evolution in technology, hackers are also using advanced techniques to hack smart vehicles. There are several entry points to smart vehicles which are listed in Figure 8.

#### 4.1.1. OBD-II Port

This port is used for monitoring various details such as emissions from vehicles, speed, mileage, etc. OBD-II ports are considered the weakest link in the vehicle since the attacker may collect diagnostic data easily and subsequently get success in accessing the in-vehicle network and deployment of malicious programs. Two types of attacks are possible on the OBD-II port, namely an in-vehicle network access attack and a Dongle exploitation attack. In the former attack type, the attacker may utilize an OBD-II port for installing the malicious device in the in-vehicle network with the main objective of obtaining physical access. In the later attack type, dongles are fitted in the OBD-II ports. These dongles can be remotely handled and decrypted by the attacker [31].

#### 4.1.2. USB and Charging Ports

Severe security threats are posed by the use of USB ports in the vehicle. Several examples of severe security threats are reprogramming of the controller processor, installation of several types of malicious codes, network card tampering, and changes in operating system functionalities. Additionally, the malicious codes inside a USB pen drive or CD can be used to hack the infotainment system. After hacking the infotainment system, hackers can easily control other parts of the vehicle such as the braking system and engine control system [32]. During the charging mechanism, Electric Vehicles (EV) are susceptible to several attacks via charging infrastructure. Additionally, the smart grid may be attacked by utilizing a charging system [33].

#### 4.1.3. Tire Pressure Monitoring System (TPMS), LiDAR and Keyless Entry Ports

The attacker can use the TPMS for eavesdropping attack to get access to the vehicle network and perform malicious activities. LiDAR and cameras are opening the door for signal jamming attack. For keyless entry attack, the hacker tries to intercept the signal for further capturing and re-directing purposes. There does not exist any adequate mechanism to protect the radio signals hence radio signals transmitted from vehicles’ keys can be easily captured by hackers. This area is open to researchers [34].

#### 4.1.4. Buse Network Ports

There does not exist a communication protection mechanism for CAN. This protocol reflects a broadcast nature and therefore each node is intended to receive the frame. This frame is not secured by either MAC or digital signature. Confidential data can be stolen or manipulated in this protocol. The hacker can send fake frames to each node and thereby vehicle may start showing unintended behavior [35].

#### 4.1.5. Vehicular Communication Ports

All the smart vehicles are enabled with Bluetooth with a range of 10 m. The mobile phone can easily get connected with infotainment as well as telematics systems for performing a range of activities such as making calls, streaming music etc. Through Bluetooth, hackers can get full access to vehicles to perform malicious activities [36]. Almost all smart vehicles are enabled with wi-fi. These smart vehicles can be connected to the internet through roadside wi-fi hot spots. The low-security level at the wi-fi hot spot may expose the vehicle to several threats since wi-fi hot spots may have outdated security mechanisms for the connection and hackers can easily target the vehicles through these weak access points [37].

Dedicated Short Range Communication (DSRC) is one of the onboard units. It utilizes radio frequency for communication. It supports short-range communication for the vehicle to infrastructure as well as vehicle-to-vehicle communication. The hacker can easily get access to the vehicle through DSRC and may perform severe undesirable activities [38]. Almost all smart vehicles are equipped with cellular technologies (3 G/4 G/5 G, etc.). These smart vehicles are now capable of vehicle-to-infrastructure as well as vehicle-to-vehicle communication with distances of several miles. hackers can perform two types of attacks, namely jamming and eavesdropping on the cellular networks [39].

### 4.2. Corrective Mechanisms to Port Threats

Several threats due to OBD-II ports can be handled by designing the framework for frame injection tracking which are coming from the OBD-II port. The second strategy can be used for firmware updates by encryption and message signing. The threats due to USB ports can be handled by designing the standard USB security framework since there exist large applications of USB devices and thereby posing severe security vulnerabilities. There are two ways to provide protection against threats due to USB ports; in the first approach, a security certificate should be required by the USB whenever it tries to connect with the internet and subsequently this certificate may give permission to link to the vehicle. In the second approach, the malware/viruses are prevented to access the restricted security area through the USB port. The threats due to electric vehicle charging ports can be handled by utilizing three schemes, namely secure firmware updates, cryptographic signatures, and authentication schemes. To protect the electric vehicle charging infrastructure, Open Charging Point Protocol (OCPP) has been developed for securing charging systems in the smart grid [40].

## 5. Comprehensive Survey of Security Solutions for In-Vehicle Network

### 5.1. Related Existing Surveys

The rapid development in advanced automotive technologies leads to prominent growth in smart vehicles manufacturing. The speedy transition towards advanced smart vehicles opens the next door to several security threats therefore in recent years, the researchers are showing more interest in designing new security frameworks for automotive protocols and particularly focused on the CAN. A large number of surveys have been published recently considering security aspects on in-vehicle network. A comprehensive survey is presented by Zeng et al. [41] considering all five protocols used in-vehicle networks. For analyzing the performance of the protocols, they assume specific parameters such as fault tolerance, cost and data transmission ability. Security threats along with appropriate solutions are discussed in this comprehensive survey covering authentication schemes, and physical controlled approach, etc. Further, in [42], a review is presented on solutions for CAN message authentication. Additionally, they analyzed the main requirements needed with aligned to the industry. Next, for improving the CAN security, a survey of proposed solutions is presented in [43]. The survey of proposed solutions also reflects the readiness and maturity for applicability in industry. For increasing the users’ safety and comfort a large number of applications have been developed but on the other hand these advanced applications pose new security threats.

Furthermore, Avatefipour et al. [44] presented a survey for describing the current limitations of CAN-bus protocol. They discussed the various different solutions for ensuring the secure communication thereby overcoming the CAN bus limitations. Additionally, a survey of attack scenarios and corresponding solutions for CAN bus is described in [45]. The survey presented the security vulnerabilities in detail owing to absence of authentication and encryption schemes for CAN bus. The survey reflected the fact that transition in technological development of automotive industry is primarily based on the CAN without considering the security factor carefully. Tomlinson et al. [46] presented the survey for CAN intrusion detection. The survey discussed the several methods for intrusion detection while considering all challenges in terms of requirements and practicability. Huynh et al. [47] reviewed several approaches for the protection of automotive applications. The main aim is to recognize the potential gaps for further improvement in performance of automotive applications. Intrusion detection systems are considered as prominent solution for protecting the CAN bus from malicious attacks.

Young et al. [48] presented a very deep survey on IDS approaches. In [49], a detailed survey of security mechanisms is presented with focus on specifically on cryptography and IDS. Wu et al. [50] presented the characteristics and constraints for designing the intrusion detection system (IDS). The detailed survey consists of the several different proposed IDS designs with corresponding limitations. Lokman et al. [51] demonstrated the deep analysis on IDS approaches. For this deep analysis, specific criteria are considered such as statistics, machine learning, frequency, and hybrid model. Bozdal et al. [52] provided the detailed outlook on security issues for CAN bus. They also presented the security vulnerabilities along with attack surface and appropriate corresponding different solutions. In [53], a review is presented with focus on three-layer security framework. The three layers considered are control, communication and sensing level. A detailed analysis is conducted considering attack perspective on security frameworks with focus on enhanced user reliability. Sun et al. [54] presented a broad survey on the security issues and challenges. The survey further illustrates corresponding defense approaches for ensuring security. Karopoulos et al. [55] presented complete survey on in-vehicle IDS. All existing in-vehicle IDS are analyzed and further classified. This paper presents the detailed analysis of security mechanisms on in-vehicle communications.

### 5.2. Security Threats to In-Vehicular Protocols and Countermeasures

**Figure 9:**
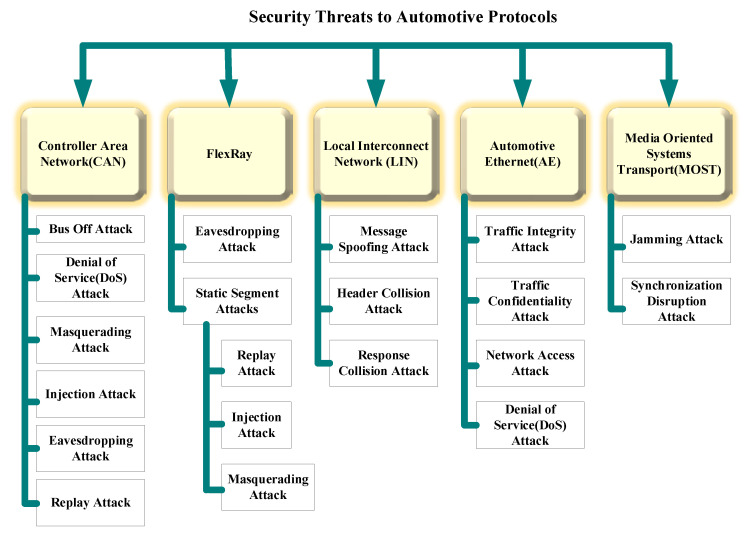
Classification of automotive protocols and possible security threats.

Classification of automotive protocols and possible security threats are illustrated in Figure 9. This classification is used as based on further discussion in this subsection.

#### 5.2.1. CAN-Centric Security Threats

Research reports reported on six types of attacks on CAN bus systems, namely bus-off attacks [56], denial of service (DoS) [57], masquerading, injection, eavesdropping, and replay attacks [58]. The attackers may get knowledge of the CAN frame since CAN frames are generally not encrypted and fail to support message authentication thereby attackers may get entry to the network easily, this type of attack is known as a Masquerading attack. Additionally, the broadcasted vehicular CAN messages may be eavesdropped on by the attackers and subsequently, they may break into the in-vehicle networks, this type of attack is known as an eavesdropping attack. Next, the attackers may try to place false signals in the bus system of the vehicle. Through OBD-II ports, the attackers may successfully establish a connection with the in-vehicle system and consequently may try to compromise the ECUs, this type of attack is known as an injection attack. Further, the vehicle’s operation in real-time may be hindered by the attacker by constantly re-sending the legitimate frames, this type of attack is known as a replay attack. Furthermore, the attacker may constantly send bits in the identification field and other fields also. This type of attack is known as a bus-off attack. Besides, the attacker may disrupt the normal processing of the in-vehicle communication by constantly delivering the CAN packet with high-priority that block valid packets of low-priority and may take control of the vehicle, this type of attack is known as a DoS attack. The first general guideline to guard against these attacks is to use encryption and authentication of the messages exchanged between ECUs [59].

#### Survey of Security Solutions Based on Machine Learning Algorithms

**Figure 10:**
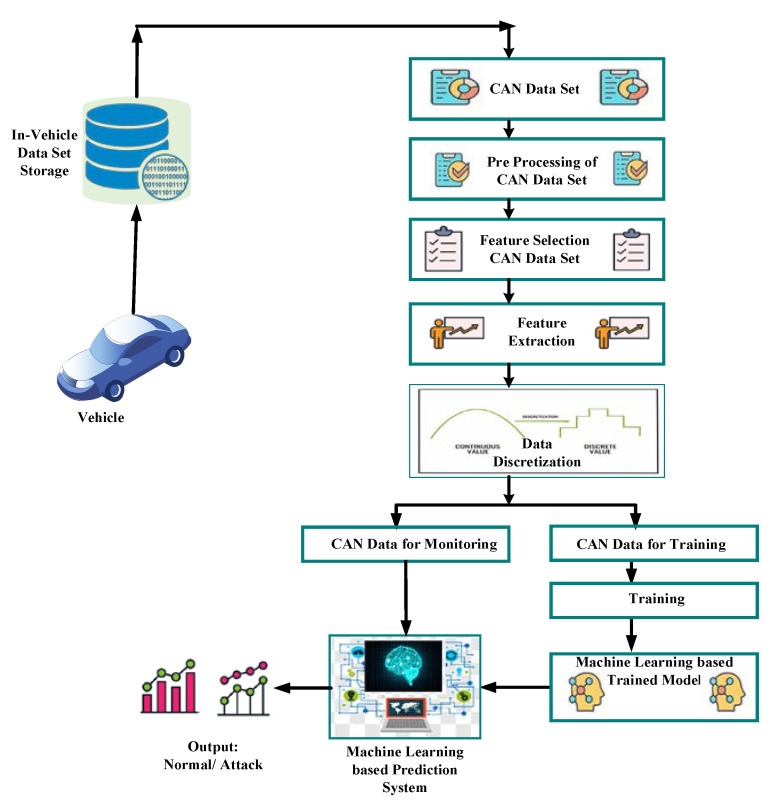
General Flow Diagram reflecting series of steps for intrusion detection using machine learning model.

In today’s world of wireless networks, machine learning (ML) approaches are considered as the most promising choice for handling security-related issues. Researchers are proposing solutions utilizing ML to deal with vehicle security issues. machine learning (ML) approaches have significant advantages as compared with other mechanisms, one of the main important features is to get optimal predictions about several types of attacks. Figure 10 reflects the use of machine learning model in intrusion detection.

For in-vehicle networks, Song et al. [60] designed an efficient intrusion detection framework. Time intervals-based analysis is conducted on the CAN messages in this lightweight framework. The analysis is started by capturing CAN messages from the CAR and performing message injection attacks. The significant insights have been derived in this analysis as attacks on the CAN traffic can be detected by carefully deep analysis of time intervals. Results revealed the fact that this lightweight framework has not been suffering from false-positive errors while detecting all of message injection attacks. Next, Kang et al. [61] designed the deep neural network-based intrusion detection framework for enhancing the security level. From the in-vehicle network packets, feature vectors (probability-based) are extracted and DNN model is trained with these feature vectors. After training, the DNN model can easily discriminate the attack and normal packets and thereby any malicious attack on the vehicle can be identified. The detection accuracy of the proposed framework is much improved as compared to traditional AI-based systems since the parameters are initially initialized via unsupervised approach of deep belief networks (DBN).

Further, Ghaleb et al. [62] proposed machine learning-based model for misbehavior detection. To detect the misbehavior effectively, regularly new features are updated and derived representing the misbehavior, status of communication. Next, historical data consisting of both normal and attacked traffic data is utilized for training the misbehavior classifier based on feed forward and backpropagation techniques of Artificial Neural Network. Jagielski et al. [63] designed intrusion detection methods using machine learning and physical-based constraints. They provided an extensive analysis of attacks targeting the adaptive cruise control, and the local sensors, namely RADAR, LiDAR, etc. The analysis of attacks revealed the fact that these attacks have a high impact on safety, comfort, and efficiency, etc. For in-vehicle networks, Seo et al. [64] designed intrusion detection system utilizing deep learning approach. For detecting the unknown attacks, the proposed framework only utilizes the normal data. Additionally, Ferdowsi et al. [65] designed the deep reinforcement learning-based system to enhance the robustness of dynamics control for the autonomous vehicles against cyber physical attacks. Game-theoretic environment is utilized for the analysis of the vehicles’ reaction to cyber physical attacks in this proposed framework.

Zhu et al. [66] designed an efficient intrusion detection framework utilizing distributed long-short-term-memory. The complexity is reduced in the detection mechanism since only binary CAN messages are utilized in this framework and thereby semantics of messages are not revealed which is a complex procedure. The proposed framework has multidimensional nature since data as well as time dimensions are taken into consideration for detection based on LSTM. The results showed high accuracy in detection. Furthermore, Eziama et al. [67] conducted a comparative analysis considering total five machine learning approaches, namely K-Nearest Neighbor, Linear and Radial Support Vector Machine, model based on Decision Tree, model based on Naive Bayes, and finally, a model based on Random Forest. To distinguish honest as well as malicious data, the recommendation system utilizes different communication nodes. The trust computation measures have been utilized to authenticate the performances of these five models, such as Recall and Precision, Receiver Operating Characteristics (ROCs).

Next, Sherazi et al. [68] proposed an Intrusion Detection framework utilizing Q-learning and fuzzy logic specifically for countering Distributed Denial of Service attacks. The simulations results attest the fact the proposed framework perform well for providing defense against Distributed Denial of Service attacks. Additionally, Khanapuri et al. [69] designed security framework utilizing convolutional neural network and deep neural network. The various sensors (RADAR, LIDAR, etc.) fitted with the smart vehicles play a major role in providing real time sensor data such as relative speed of the neighbors, as well as range, etc. These noisy sensor data are followed a Guassian Distribution and then finally utilized for training the convolutional neural network and deep neural network. Furthermore, Song et al. [70] designed a deep convolutional neural network-based effective intrusion detection framework for enhancing the protection level of the CAN bus. In the proposed framework, deep convolutional neural network model easily identifies the malicious traffic after properly learning the pattern of the traffic data. The proposed framework provides a high detection performance with significant reduction in complexity. Xiao et al. [71] proposed lightweight security framework to counter against attacks on the CAN bus through various accessing points. The proposed security framework is divided into two individual frameworks, namely simplified attention (SIMATT) framework based on the machine learning and second one known as security control unit (SECCU) framework. The use of two individual framework instead of single one reduces the computational cost significantly.

Next, Guo et al. [72] proposed security framework consisting of two parts, namely designing of context-aware trust management model as well as reinforcement learning model. The first model aimed at evaluating the trustworthiness of messages. The second model is used to select the appropriate evaluation strategy in order to ensure the high precision in evaluated results. Katragadda et al. [73] designed sequence mining methodology for detecting low-rate injection attacks in CAN. The efficacy of the proposed approach is measured by gradually changing characteristics of the attacks. Additionally, Rasheed et al. [74] proposed deep reinforcement learning-based framework for maximizing the robustness of control for autonomous vehicle. In this environment, the attacker tries to manipulate the sensor readings of the vehicle for disrupting the optimal working mechanism of autonomous vehicle on the road. By manipulating the sensor readings such as safe distance, etc., accident of autonomous vehicles may happen. Lin et al. [75] proposed effective intrusion detection framework utilizing deep learning for the three specified attacks known as impersonation, Denial of Service, and fuzzy on the CAN traffic. The proposed framework reflects good efficacy in handling premature convergence by using evolutionary optimization algorithm. The results proved the outstanding performance of the proposed framework.

Besides, Hossain et al. [76] proposed Intrusion Detection framework utilizing Long Short-Term Memory for providing protection against CAN bus attacks. First, attack free normal data is extracted from the CAR for generating the genuine data set subsequently attack data is extracted by injecting attacks. This real time genuine data set is used in training the LSTM-based model. Additionally, Angelo et al. [77] proposed an intrusion detection framework utilizing two algorithms. First algorithm aimed at learning the behavior of the traffic data while second is data-driven algorithm. These two algorithms are used to focus on real-time classification of traffic data resulting into prior alert towards the presence of malicious messages. Further, Table 2 illustrates the summary of in-vehicle security solutions based on machine learning algorithm.

From the above extensive research, we get the crucial facts such as the use of machine learning approaches are considered as prominent solution for detecting and predicting several types of attacks on in-vehicle network. Therefore, machine learning-based frameworks are used for analyzing CAN traffic effectively. The effectiveness of machine learning-based approaches is based on several factors. The first crucial factor is the pre-processing methodology adopted for pre-processing the raw CAN data. This is considered as crucial factor since no specification is provided by the automotive manufactures on decoding the raw data features. Supervised machine learning-based approaches are quite time consuming due to labelling of raw CAN data, identification and classification CAN attacks, etc. On the other hand, unsupervised machine learning-based mechanisms use data for finding the common patterns and further utilizing these patterns for classification of CAN traffic and identifying the anomalous behavior.

#### Survey of Security Solutions Based on Cryptography Techniques

**Figure 11:**
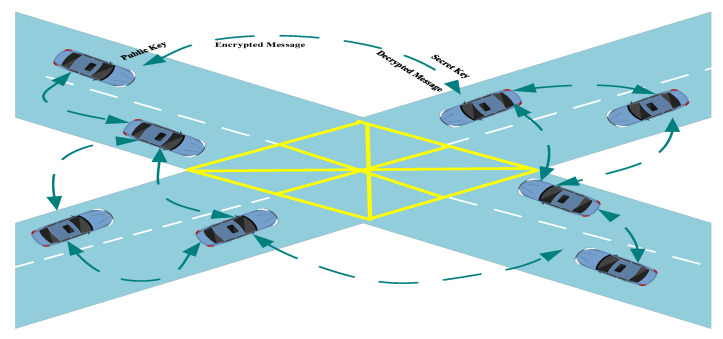
Use of general asymmetric cryptography approach for securing vehicle to vehicle communication.

Cryptography algorithms are used to counter the diversified cyber-attacks on the in-vehicle network of smart vehicles. Several security frameworks have been proposed using cryptography algorithms. The hackers are using real time latest advanced techniques to attack on the vehicles and therefore, no standard security framework is available with guaranteed resolutions for latest threats. Researchers are using new advanced cryptographic algorithms in designing security framework to provide protection to CAN bus and thereby protecting the data frames from manipulation. Figure 11 depicts the general asymmetric approach used in securing the vehicle-to-vehicle communication.

Nilsson et al. [86] proposed a data authentication framework for modification and injection attacks. The proposed data authentication is known as delayed authentication since MAC is derived on a compound of successive messages and sent with other subsequent messages. Next, Herrewege et al. [87] explored the implementation issues of the message authentication protocol for CAN bus. After successful investigation, they find out various constraints which are related with backward compatible message authentication protocol and presented a new message authentication protocol in order to address the existing constraints. Additionally, Hazem et al. [88] designed a new protocol known as message source authentication protocol. The proposed authentication protocol performs well with minimum overhead. The implementation of the proposed protocol does not require neither any modifications in hardware for CAN network nor any changes in existing CAN message sets. Further, Groza et al. [89] utilize symmetric primitives for designing the authentication protocol having two main mechanisms, namely mixing of MAC and splitting of keys. In the proposed protocol, authentication keys are split among multiple groups of nodes results in progressive authentication as compared with traditional approach of authentication for each node independently.

**Figure 12:**
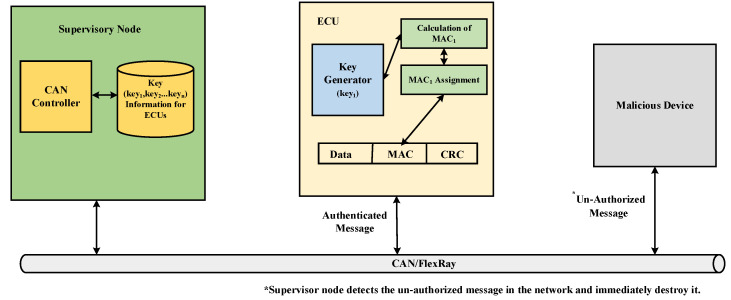
Centralized framework consisting of Supervisory Node [92].

In [90], several different methodologies are presented for preventing the unauthorized data transmission thereby increasing the security level in CAN and in [91], protocol is presented to counter against DoS attacks. Additionally, a secure channel is provided by the proposed protocol between external devices and in-vehicle network components. The proposed protocol consists of two main authentication processes, namely checking the authenticity of transmitter and data validation through message authentication. Further in [92], a centralized framework is presented for CAN with enhanced CAN controller. The monitoring system in the proposed centralized framework utilizes a message authentication code. The mechanism starts by authenticating each ECU in the bus by a node and then message authentication codes are reviewed which are assigned to messages being transmitted in the bus. Figure 12 illustrates the working of centralized framework in detail.

Next, in [93], an advanced framework is proposed which is based on runtime verification to provide security against several attacks on CAN. The proposed framework uses copilot method for performing run time detection. Additionally, in [94] a CAN authentication protocol is presented for providing immunity against attacks. The proposed lightweight authentication protocol is effective against a DoS attack. The proposed authentication protocol has three main stages and through these stages all weak points are addressed to provide secure and robust environment for CAN. Further, authors in [95] designed a security framework for providing security in CAN. For the security of transmitted messages, the proposed framework utilizes a truncated MAC. Additionally, in a data frame, it utilizes a segment of MAC. Therefore, the proposed framework is using two different mechanisms to secure the CAN against malicious attacks. The results attested the fact that the proposed framework effectively handle the replay and tampering attacks. Furthermore, new methodology based on cryptographic techniques is presented in [96] for increasing security in CAN. The proposed lightweight framework uses stream cipher to encrypt messages while protection against external attacks is provided through a key management mechanism. The results shown that the proposed framework is characterized with two main features such as minimum memory requirements as well as high efficiency as compared with other MAC-based schemes. Further Table 3 illustrates the summarized key points of literature representing security solutions based on cryptographic techniques.

From the above extensive research, we get the crucial facts such as the use of cryptography approach against security threats to in-vehicle network has all potential advantages except that the CAN bus controller requires additional computational resources. Generally, there are two main components in the Cryptography approach. First is known as Message Authentication Code (MAC) and another is called as cryptosystems having two fields symmetric and asymmetric. Further, Integrity and Authentication is ensured by the MAC while confidentiality is provided by the symmetric and asymmetric cryptosystems. Additionally, session keys can be utilized for providing authentication. For vehicle safety, the load on the CAN bus and latency issue in response time should be within specified limit. Additionally, error detection in data frame transmission is provided by the Cyclic Redundancy Code (CRC) at CAN bus. ECUs are having their own limitations in terms of computational capacity thereby lightweight encryption is one of the solutions for handling this issue since ECUs are core components inside the vehicle handling various functions simultaneously. The bus may be heavily loaded during key exchange and pre-loaded keys in the ECUs can tackle this situation in key distribution environment. The Hardware Security Module (HSM) in ECUs can be effectively utilized for performing encryption and decryption in optimal time and compensating the issue of resource constrained ECUs. Although several significant developments towards security framework based on cryptography algorithms can be seen but cost factor in successful implementation of these schemes cannot be ignored.

#### 5.2.2. Security Threats—FlexRay

Eavesdropping [115] and static segment attacks [116] are the two main types of threats in FlexRay. In the case of the former type of attack, FlexRay messages are accessed by the attackers and consequently, the attacker can obtain all the critical information. This attack results in leakages of data, impact on data confidentiality, and also security concerns. In the case of later types of attack, the communication cycle of FlexRay having a static segment is attacked. This attack also includes replay, injection, and masquerading types of attacks. The preventive mechanism for both these types of attacks consists of the implementation of an advanced scheme for authenticating the message within the static segment [117]. Timed Efficient Stream Loss-tolerant Authentication (TESLA) [118] is the authentication protocol.

#### 5.2.3. Security Threats—Local Interconnect Network (LIN)

Three attacks usually occur to LIN, namely message spoofing [119], header collision, and response collision attacks. In the message spoofing attack, the attacker tries to interrupt vehicular communication by sending false unauthorized messages with the only objective of shutting down LIN. This attack is caused by the vulnerabilities in the master-slave model of LIN. In the collision response attack, the hacker tries to exploit the error handling protocol of LIN. In this attack, along with a valid message, the attacker sends an illicit message consisting of a false header concurrently. Consequently, the message transmission is stopped by the legitimate slave node immediately, whereas illegitimate messages will be accepted by all other nodes. In the header collision attack, the hacker tries to create a conflicting situation. The attacker sends an incorrect header to create a conflicting situation since a valid header from the master node is also present in the system. According to the valid header, the response should be released by the specified slave node, on the other hand, an incorrect header states that the changes occur in the source node. This attack may create several life-threatening unwanted functions for the example steering wheel of the vehicle can be locked while the vehicle is driving on the road, opening the sliding doors of the vehicle and much more, these functions not only cause threats to the life of passengers but also damage the overall vehicle. In the corrective mechanism against these types of attacks, the slave node can send unusual signals for overwriting the fake messages of the hacker whenever the value of the bus mismatches from its response [120].

#### 5.2.4. Security Threats—Automotive Ethernet (AE)

Four types of attacks usually occur to Automotive Ethernet such as traffic integrity, traffic confidentiality, network access, and DoS attacks [121]. In the network access attacks, the hacker first establishes a connection with the unsecured port of the switch and then via this connection hacker tries to connect to the Ethernet network. The ultimate objective of the attacker is to get access to the network and subsequently take control over several different nodes or control the network remotely. In the traffic confidentiality attacks, the attacker first gets access to the network and then he attacks the network and tries to overhear activities in the network. The traffic integrity attacks can be considered similar to attacks as a man-in-the-middle. In this type of attack, information is exploited by diverting the traffic towards the compromised node. There are two types of these attacks, namely session hijacking as well as replay attacks. DoS attacks are classified into two categories in Ethernet. In the first category of DoS attack, the attacker tries to disrupt the Ethernet infrastructure and convert it totally into an unusable form. In this attack, the attacker first physically destroys links or hardware. Next, the second category of DoS attack is also known as resource depletion attacks or protocol-based DoS attacks. In this attack, the attacker constantly submits frames for analysis in order to waste energy. The corrective mechanism should consider the authentication, and frame replication along with the virtual local area network segmentation scheme [122].

#### 5.2.5. Security Threats—Media-Oriented Systems Transport (MOST)

Two types of attacks which are generally occurred to MOST, namely jamming and synchronization disruption attacks. In the synchronization disruption attacks, the hacker tries to tamper with the synchronization of MOST by sending the fake timing frames continuously. In jamming attacks, the hacker tries to interrupt low-priority legitimate messages having specified length by continuously delivering misleading messages. Additionally, the hacker may continuously request data channels on MOST transmission via control channels. The corrective mechanism has three main approaches such as source node authentication, exchanged messages should be encrypted, and strict enforcement of firewalls and gateway [123].

## 6. Multi-Layered Security Framework

**Figure 13:**
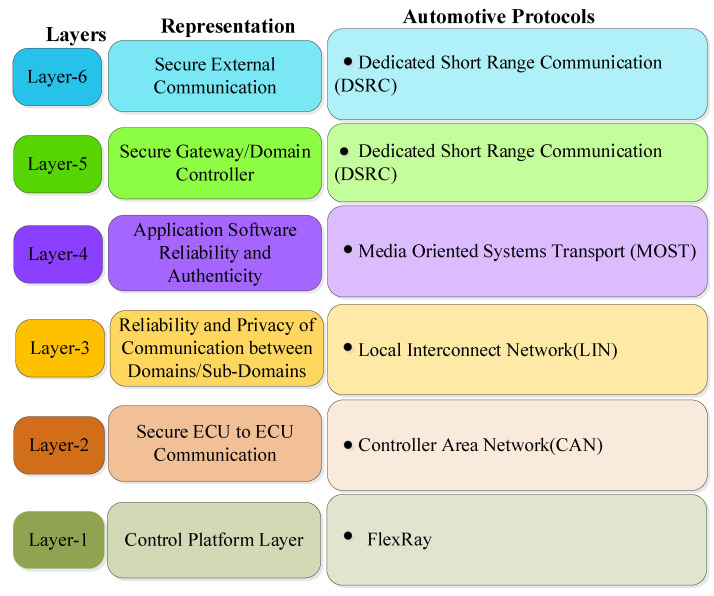
Multi-layered Security Framework for in-Vehicle Network.

The modern advanced vehicles have complex and sophisticated in-vehicle architecture. Additionally, these vehicles are equipped with highly sensitive sensors, different electronic devices, computer systems, etc. in order to secure these sophisticated systems, a coordinated, systemic integrated cybersecurity framework is needed to design the solutions and to minimize security risks. For the cyber security of in-vehicle networks, a multi-layered approach is the need of the hour. We need an integrated security approach since hackers invade vehicles through cyber and via the physical world. Further, Figure 13 illustrates the multi-layered security framework for in-vehicle networks with corresponding automotive protocols. In the context of scientific novelty, it is highlighted that the multi-layered security framework is proposed based on the findings of critical investigation of existing literature. Each layer addresses a specific type of security threat for in-vehicle communication network. It is highlighted that the multilayer security framework is proposed based on the understanding and knowledge aided by critical analysis and findings of existing approaches for the security of in-vehicle network. We found that the literature is lacking in terms of cohesive multi-layer security framework for in-vehicle network. In this framework, we addressed different security issues of in-vehicle network in specific layers including ECU-boot level security, ECU to ECU communication, domains/sub-domains communication, application software update or version issues, gateway security controller, and vehicle to outside services.

However, it is clarified that in the proposed integrated multilayer security framework, each layer ensures specific functionality and address security threats. This is the reason we divided the framework into six layers staring from ECU-boot level security, ECU to ECU communication, domains/sub-domains communication, application software update/version issues, gateway security controller, and finally in-vehicle network to vehicle outside services security issues. In the Secure Gateway/Domain Controller layer, we are more concerned about security of gateway while at the secure external communication layer, we have a priority to protect the communication channels. In terms of in-general functional roles all automotive protocols are somehow or other play role in all layers due to complex structuring of modern in-vehicular system. However, if we make deep analysis based on maximum effectiveness of automotive protocols in terms of providing functionality to domain/sub-domain of in-vehicular system, then we can identify which specific automotive protocol is more suitable to a layer. Therefore, we used a protocol for a layer with more effective and suitable functionality consideration in mind while developing the framework.

### 6.1. Control Platform Layer

This layer aims at enhancing the security of platform. This layer can be assumed as vehicle main nerve center for securing the in-vehicle network of smart intelligent vehicle against malicious threats. Control layer aspect deals with security solutions for protecting firmware of ECU, enhancing security at booting level, and also security of hardware modules (HM). The original equipment manufacturer (OEM) trusted server and trusted platform module (TPM) chip at ECU play a major role in this mechanism. The secure connection is effectively maintained between these two modules for the exchange of encrypted firmware update image file and data for enhancing security at booting level, ECU firmware, etc. The ECU firmware should be updated frequently to counter the diversified attacks in current advanced technological automotive era. Further, authenticity of ECU firmware update can be verified via signing/secure flashing mechanism.

### 6.2. Secure ECU to ECU Communication in In-Vehicle Network

This layer ensures message delivery with integrity proof between ECUs. The hackers are targeting a large volume of sensitive information which is actually generated by The ECUs. The security mechanism should ensure the reliability, confidentiality, and integrity of this sensitive information. A hardware security module (HSM) chip should exist in the modern in-vehicle network that work as security controller. The security controller has three main modules, first module is responsible for ECU id authentication and ECU state verification, second module handles encryption/decryption of messages exchanged and third module control the secure flash storage. All messages from the ECUs should be forwarded to HSM chip (security controller). The security controller will analyze the messages and identify the destination ECU. Based on the state of the destination ECU and message security properties, it decides whether the message should be forwarded to the destination ECU or not.

### 6.3. Reliability and Privacy of Communication between Domains/Sub-Domains in In-Vehicle Network

This layer ensures the reliability and privacy of communication between domains/sub domains in the vehicle. A vehicle domains/sub-domain reflects the grouping of functions and systems with respect to specified areas, for example telematics domain, infotainment domain, chassis domain, powertrain domain, sensor domain, and body domain, etc. This layer protects the domains/subdomains by using four security mechanisms as follows:

○ Message authentication system: Cryptographic certificate is used to ensure an authenticated sender and message integrity. This certificate is added to all messages in the network.
○ Encryption: Messages are encrypted inside the vehicles to guard against loss of data and identity theft since messages are distributed with several different ECUs.
○ Detection of intrusion: The corrective mechanism should use the cryptographic accelerators and security subsystems integrated with the microcontroller to guard against threats.
○ Validation at ECU level: In the vehicle network, ECU’s validity is checked first at the start of the engine and also afterwards at specified time intervals.

### 6.4. Application Software Reliability and Authenticity

All application software should ensure reliability and authenticity qualities. The hackers are using software download/update features for attacks. The secure mechanism immediately updates the vehicle software whenever any security vulnerabilities is detected. The application software is generally developed by the third-party manufacturer and verified software file image is sent to original equipment manufacturer (OEM) trusted server for uploading. The software update manager module at OEM trusted server sends notification to vehicle owner for the availability of updated software version. After confirmation from the vehicle owner, the updated software version is installed in secure in-vehicle network storage area. In this entire mechanism, security of channels is a prime concern. There are various techniques exists for enhancing the security of channels, namely hash function, digital signatures, blockchain technologies, etc. Additionally, for verifying the trustworthiness of the encryption mechanism, modern advanced microcontrollers have features such as integrity testing in real time, etc.

### 6.5. Secure Gateway /Domain Controller

This layer ensures the security of gateway/domain controller. All modern critical gateways must be equipped with next generation advanced security mechanisms such as advanced key management schemes/firewalls, intrusion detection schemes, etc. The main responsibility of the gateway is to maintain the secure configuration of the system. Context-aware routing is implemented by the gateway, in this routing mechanism, the gateway checks the validity of the messages, and all valid messages are permitted to transfer via the gateway to the corresponding destination through a different number of complex controls. The automotive manufacturers are working towards designing robust and secure gateway controller module. The cryptographic credentials are stored in the secure hardware extension (SHE) chip which is the part of secure gateway controller module. The main role of SHE chip is to protect the cryptographic credentials from the hackers. The public key infrastructures (PKI) of various communication service providers manages the cryptographic credentials inside the SHE chip.

### 6.6. Secure External Communication 

This layer ensures secure communication from in-vehicle network to various services outside the vehicle. This layer provides authentication and message validation functionality for protecting message integrity and thereby protecting the communication channels from data manipulation and data theft. Due to these features, vehicle-to-everything communication, telematics, etc. are secured. All communications from in-vehicle communication stack to roadside unit (RSU) must be established via trusted communication authority (TCA) for providing authentication and message validation. Certificates which are used during communication between in-vehicle communication stack and TCA, further between TCA to RSU should be changed frequently for maintaining the integrity of the sender. Additionally, the integrity of the message content can be protected with the help of digital signature.

## 7. Open Challenges and Future Research Directions

**Figure 14:**
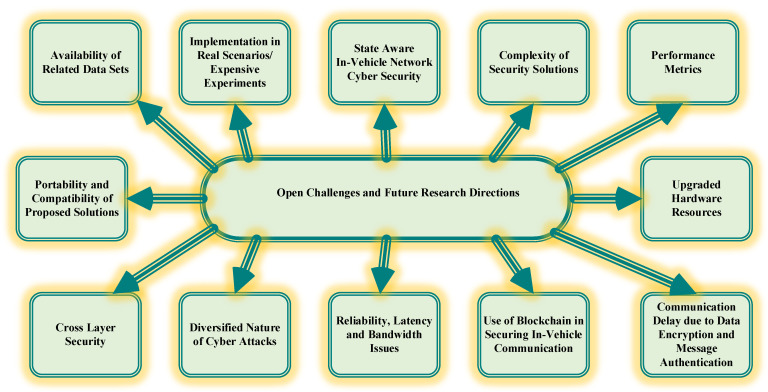
Open challenges for security of in-vehicle network.

The rapid development in vehicular communication paves the way for more threats. There is an urgent requirement for the development of security mechanisms to counter these threats. The diverse cyber-attacks on in-vehicle network of smart vehicles may lead to damage to the vehicle as well as possible loss of life. All stack holders should consider the security of in-vehicle networks a high priority. The below section will discuss in detail various challenges and future research directions for in-vehicle network security aspects [124,125]. Figure 14 illustrates the open challenges for in-vehicle network.

### 7.1. Availability of Related Data Sets

The impact of advanced research on the security of vehicles depends on the genuine real-time data sets containing message semantics for experimentation. Unfortunately, the availability of related genuine datasets is the main constraint in advanced research. The CAN bus datasets are the intellectual property of particular automobile manufacturers and therefore these data sets are not available publicly for experimentation. The possible solution to this issue is a collaboration between automobile manufacturers and the researcher. Further, data frames in the in-vehicle networks are generated in milliseconds therefore labelling of data is a complex issue. Although predefined attacks are identified by supervised learning, we have to focus on developing security solutions having the capability of handling new and diversified attacks [124].

### 7.2. Implementation in Real Scenarios/Expensive Experiments

The research advancements for countering cyber-attacks on smart vehicles should be implemented in real scenarios for achieving optimum results. However, the implementation of research solutions in real scenarios has various limitations. A researcher cannot afford to buy a car for conducting experiments. The seamless solution in these circumstances is collaborative efforts between research organizations and automotive manufacturers [125].

### 7.3. State Aware In-Vehicle Network Cyber Security

Some security approaches do not consider the different vehicle states. Therefore, the efficacy of the security approaches is only limited to specified vehicle states. The security mechanisms should be robust in nature with higher coverage to different vehicle states [116].

### 7.4. The Complexity of Security Solutions

Researchers are proposing a variety of security solutions based on AI/machine learning, neural network, cryptography, etc. The performance of the proposed security solutions can be increased by combining different backbone techniques used in developing security solutions. Several vibrant factors can be considered while developing security solutions such as computational requirements in the proposed security solution should be low [117].

### 7.5. Performance Metrics

The performance of the security solutions can be observed concerning a variety of metrics such as accuracy, recall, precision, F-score, etc. The proposed security solutions should have improved false-positive percentages for detecting attacks. In the real-world scenario, there is a need of developing security solutions having autonomous actions in terms of indicating and mitigating attacks [118].

### 7.6. Portability and Compatibility of Proposed Solutions

The proposed security solutions should have portability and compatibility features, this can be achieved by testing the performance of the proposed security solutions in real-time scenarios. The proposed security solutions should also qualify for the minimum standard mark as per the cyber security standards [119].

### 7.7. Upgraded Hardware Resources

The advanced security solutions need upgraded hardware resources used on in-vehicle networks. There is a need for scalable hardware resources in in-vehicle networks for supporting new functionality. Research is going on developing next-generation ECU’s and gateways for identifying the suspicious messages and thereby stopping the transmission of these fake messages. In the network [120].

### 7.8. Cross-Layer Security

The advanced security solutions are more efficient enough than the traditional approaches since they focus on cross-layer security for providing secure data communications for in-vehicle networks. The traditional security solutions either focus on physical layer or application layer security which is why their efficiency is limited [121].

### 7.9. Diversified Nature of Cyber Attacks

Researchers are now working on developing security solutions to counter as many attack patterns as possible. The rapid development of smart vehicles gives rise to advanced security issues as well. The next-generation security solutions will protect against manipulation of CAN frame semantics [122].

### 7.10. Use of Blockchain in Securing In-Vehicle Communication

Future research directions towards securing CAN bus of in-vehicle networks should include blockchains. Blockchain technology is new technology and has the capability to combat cyber-attacks. Blockchain is defined as a distributed data structure. The blocks in the distributed data structure are in chronological order and chained cryptographically. The main security challenge in utilizing blockchain technology for securing in-vehicle systems is a consensus mechanism. The consensus mechanism utilized in blockchain technology will have a direct impact on the security of the system [123].

### 7.11. Reliability, Latency and Bandwidth Issues

Reliability is the crucial factor for optimal performance of in-vehicle networks, for example, consider the case of ADAS data transmission, if the ADAS data transmission is unreliable then it may result in damage to the vehicle and probable life hazards to passengers. Latency is another important parameter in the vehicle network. All safety components have stringent delay specifications for ensuring optimal reliability. The third important issue is bandwidth, for high bandwidth and communication efficiency additional requirements are needed in in-vehicle networks which raises several challenges [124].

### 7.12. Communication Delay Due to Data encryption and Message Authentication

The communication delay is directly affected by the processing capabilities of the microcontroller since data encryption and message authentication require additional computational resources. Additionally, the size of the data frame is also a crucial factor in communication delay as the transmission mechanism takes additional processing time [125].

## 8. Conclusions

Manufacturing of modern smart intelligent vehicles are the result of integration of communication technologies and advanced computing with automotive industries. Initially automotive protocols are designed without considering the security threats, but current scenarios require advanced security schemes for these protocols to counter against malicious attacks. The security schemes are using different technology environments such as using cryptography techniques and machine learning algorithms. For enhancing the security of in-vehicle network, several research articles have been published that have used cryptography and machine learning algorithms for designing security frameworks and still research is going on for finding optimal solutions. Cryptography techniques generally use identifiers in data frames, finding manipulations in sending time, and message authentication, etc. for enhancing the security levels on the other hand machine learning approaches are using different algorithms to design the framework and train the model accordingly with training data. There are two aspects while developing security solutions, namely transfer layer and physical layer security solutions. Applying cryptography approach at transfer layer by utilizing message authentication code, etc. is having various constraints due to the limitation of memory, computational capability, etc. Therefore, it will be good practice to choose security solutions at physical layer. Though significant improvements can be seen in current development of automotive protocols, still there are open research issues such as security against advanced attacks, bandwidth requirements, attack detection and resolution efficiency, latency, compatibility issues, and cost, etc. There is a need of designing the efficient robust security scheme which can handle various issues of automotive protocols as well provide protection against variety of security threats. 

In this noble piece of research work, we presented a systematic survey covering the communication vulnerabilities of in-vehicle network, proposed security methods based on machine learning algorithms and cryptography techniques, characteristics of in-vehicle protocols, integrated multilayer security architecture of in-vehicle network, significant insights about improvement of the security level in in-vehicle communication. Finally, we provided the detailed discussions about futuristics potential research directions for securing the in-vehicle communication. Both sectors, including academia and the industry, have shown incredible concerns towards security of in-vehicle network therefore we are sure that this systematic survey will provide a strong base for the research and development team for acquiring valuable inputs regarding security challenges of in-vehicle network and designing enhanced solutions.

## Figures and Tables

**Table 1:** Classification of in-vehicle network communication protocols.

In-Vehicle Network Communication Protocols	Domain	Bandwidth	Salient Features	Drawbacks	Topology	Standard	Cabling	Max. Nodes Supported	Messaging
Controller Area Network (CAN)	Powertrain, Body Control	125 Kbps–1 Mbps	Low cost	Less Bandwidth	Star, Ring, Linear bus	ISO 11898	UTP	30	Multi-Master
Local Interconnect Network (LIN)	Simple Applications (Less Time Critical)	125 Kbps–1 Mbps	Low cost	Low Speed	Liner bus	ISO 17987	1-Wire Cabling	16	Master-Slave
FlexRay	Advanced Chassis Control	Up to 10 Mbps	High Speed	High Cost	Star, Linear bus, hybrid	ISO 17458	UTP	22	Multi-Master
Media-Oriented Systems Transport (MOST)	Infotainment Applications	Up to 150 Mbps	High Speed	High Cost	Ring	ISO 21806	UTP and Optical	64	Streams/Cyclic Frames
Automotive Ethernet	High Bandwidth Applications	Up to 100 Mbps	High Speed	High Cost	Star, Linear bus	ISO 21111	UTP	Based on Switch ports	Based on IP

**Table 2:** Summary of characteristics of security solutions based on machine learning algorithm.

Focused Area	Algorithm Used for Detection	Adversary Model	Robustness	Strength	Weakness	Complexity Level	Accuracy in Detection (%)
CAN Bus	Time Intervals-based Framework for Analysis [60]	Denial of Service Attack	High	Lightweight IDS	No provision of Sequence Analysis	Low	>90
CAN Bus	Deep Neural Network [61]	Intrusion Detection System	High	Effective Class Discrimination	Extensive Data Set is required for learning	High	>90
VANET Applications	Artificial Neural Network [62]	Misbehavior Detection System	High	Effective Data Analysis and Feature Extraction before building Classifier	Lack of Comprehensive Detection Mechanism	High	>90
Connected Vehicle	Physical-based constraints and Machine Learning Algorithm [63]	Manipulation of Data	Medium	Collaborative Adaptive Cruise Control Attack Analysis	Unable to find dependencies in hidden states	High	90% for velocity and Position change attack
CAN Bus	Generative Adversarial Networks based on Deep Learning [64]	Fuzzy Attack, Denial of Service Attack, Spoofing Attack	High	Real time IDS for In-Vehicle Network	Lack of Efficiency in distinguishing other type of Anomalous Traffic	High	>90
Autonomous Vehicle	Long Short-Term Memory and Reinforcement Learning [65]	Cyber Physical Attack	High	Effective extraction of temporal features using LSTM	Extensive Data Set for learning	High	Not Available
CAN Bus	Long Short-Term Memory [66]	Flood Attack, Replay Attack, Spoofing Attack	Medium	Multi-Dimensional Anomaly Detection Model	Issues of Random Weight Initializations	High	>80
Machine to Machine Communication	Five machine learning approaches, namely K-Nearest Neighbor, Linear and Radial Support Vector Machine, model based on Decision Tree, model based on Naive Bayes, and finally model based on Random Forest [67]	Trust Computation	Medium	Comparative Analysis of machine learning-based trust models	Issues in finding the optimality in trust boundaries	High	Not Available
Internet of Vehicles	Fuzzy and Q-Learning [68]	Distributed Denial of Service Attack	High	Self-Learning Capability	Unable to Provide Efficient Protection Against other Types of Attacks	High	Not Available
Autonomous Vehicle	Convolutional Neural Network and Fully Connected Deep Neural Network [69]	Platoon Attack	High	Effective Performance with Time Series Classification	Scalability Issues	High	>90
CAN Bus	Deep Convolutional Neural Network [70]	Spoofing, Denial of Service, Fuzzy	Medium	Experiment is Performed on the Real Vehicle	Semantic Features are not considered for further detecting the unknown attack	High	>80
CAN Bus	Recurrent Neural Network [71]	Impersonation, Denial of Service, Fuzzy	High	Vehicle status can be monitored in real time without domain knowledge	Issues with long sequences	High	>90
VANET	Reinforcement Learning [72]	Trust Computation	High	Model for evaluating the reliability of information	Issue of overloading of states resulting into diminishing output	High	90%
CAN Bus	Intrusion Detection based on Frequency Analysis [73]	Replay Attack	Medium	Model can be adaptable to different Automotive Manufacturer	No consideration about different vehicle states	Medium	Not Available
Autonomous Vehicle	Long-Term Short-Term Memory, Generative Adversarial Network, Reinforcement Learning [74]	Cyber Physical Attack	High	Model can Extract Features from huge data sets	No consideration for non-linear modelling with dynamics	High	Not Available
CAN Bus	Deep Learning [75]	Impersonation, Denial of Service, Fuzzy	Medium	Sequential Patterns Analysis for Detecting the Change in Traffic Behavior	No consideration for other Cyber Attacks	High	>80
CAN Bus	Long Short-Term Memory [76]	Spoofing, Denial of Service, Fuzzy	Medium	CAN data sets are collected from real Vehicle	Experiment is conducted in offline mode, no consideration for other unknown attacks	High	>90
CAN Bus	Cluster-based learning algorithm and Data Driven algorithm [77]	RPM Attack, Fuzzy Attack, GEAR Attack, Denial of Service Attack	High	Data driven model with classification based on unsupervised approach	No consideration about self adaptability feature and other attack types	High	>90
CAN Bus, Cloud-based IDS	Deep Learning [78]	Malware, Denial of Service, Command Injection	Medium	Mathematical Modelling and Testbed Experiment on Robotic Vehicle	No consideration against physical jamming threat	High	>85
CAN Bus	Deep Learning [79]	Replay, Spoofing	High	Experiment is conducted on the real data acquired from the physical vehicle	No Comparative Analysis with other Deep Learning Schemes	High	>95
CAN Bus	Deep Contractive Autoencoders [80]	Fuzzy, Impersonation, Denial of Service	Medium	Three different Vehicles are utilized for AN Data Collection and Discriminating the Anomalies.	Lack of Efficiency in distinguishing other type of Anomalous Traffic	High	>90
CAN Bus	Machine Learning [81]	Spoofing, Denial of Service, Fuzzy	High	Simulation is performed on the real data collected from licensed vehicle	Support Vector Machine underperform with more noisy data set	High	>90
CAN Bus	Long-Term Short-Term Memory and Recurrent Neural Network [82]	Spoofing	High	Authentication based on finger print signals	No provision for optimization of FPGA Accelerator	High	>95
Internet of Vehicles	Deep Transfer Learning [83]	Flooding, ARP, Impersonation	High	For New Attack type, Model can update without any labelled data requirements	Issue of Negative Transfer	High	>90
CAN Bus	Machine Learning [84]	Denial of Service	Medium	High Search Ability and Avoidance of Premature Convergence	No consideration for other Cyber Attacks	High	>90
CAN Bus	Long Short-Term Memory and Convolutional Neural Network [85]	Replay, Denial of Service, Fuzzy, Spoofing	High	Model is verified with automatic vehicle data sets	No consideration for other attacks types	High	>90

**Table 3:** Summary of characteristics of security solutions based on Cryptographic techniques.

Focused Area	Strength	Approach/Methodology	Weakness	Adversary Model	Key Aspects
CAN Bus	Delayed Data Authentication for avoiding disruption with real time traffic	CBC-MAC [86]	No provision for MAC calculation with diversified compound sizes	Spoofing, Injection	The proposed scheme utilizes compound message authentication codes for delayed data authentication. In this scheme, on a compound of successive messages, message authentication code is calculated.
CAN Bus	Backward Compatibility, no need to modify existing nodes	Counters, HMAC, Symmetric Key [87]	All nodes must know about pre-shared key before verifying the messages	Replay, Sniffing, Injection, Spoofing	The proposed scheme utilizes HMAC in designing lightweight authentication protocol.
CAN Bus	The proposed protocol can be practically deployed in the vehicle without hardware modification	Session Keys, Magic Number [88]	Issues in exchange of authentication data owing bandwidth limitations	Injection, Replay	A lightweight authentication protocol is proposed to CAN bus.
CAN Bus	Source authentication is effectively managed	LMAC, MD5 [89]	The proposed scheme works well with only lower number of nodes	Injection, Replay	In the proposed scheme, authentication protocol is designed utilizing MAC mixing and key splitting mechanism.
CAN Bus	Proposed solution is software-based and can be easily applied	Secret Keys (Symmetric Pair wise) and MAC [90]	No comparative analysis is provided for testbed experiment	Replay, Masquerade	Different parameters are used to design the scheme, namely MAC ID, Secret Keys. Transmitter and Receiver use shared secret key.
CAN Bus	Provide Secure channel for vehicle to external communication	CRC, MAC [91]	No comparative analysis is provided for other types of attacks	Denial of Service	Designing of new secure protocol for CAN.
CAN Bus	The proposed centralized security scheme is verified with FPGA board	HMAC, SHA-256 [92]	No comparative analysis is provided for key exchange environment	Spoofing	In the proposed scheme, central authentication framework is designed.
CAN Bus	The proposed scheme authenticate ECU with provision of session keys establishment	Session Keys, ECC, HMAC [93]	AVISPA tool is used for Security Validity, other platforms should be used for measuring the efficacy	Authentication of ECU in CAN for providing protection against attacks	The proposed scheme utilizes the ECC. In the adapted protocol, elliptic curves are implemented with variety of different parameters.
CAN Bus	Simulation is performed using Vector Canoe.	Authentication (Lightweight) [94]	No comparative analysis is provided for other types of attacks	Denial of Service	A new CAN authentication protocol is proposed.
CAN Bus	The proposed scheme utilize two different MAC methods.	Key Management, MAC [95]	No comparative analysis is provided for other types of attacks	Replay, Tampering	A new message authentication-based protocol is proposed for CAN.
CAN Bus	The proposed scheme requires less memory and speed is high	SHA, RC4, AES-128 [96]	No comparative analysis is provided for other types of attacks	Flood, Replay, Masquerade, Eavesdrop, Brute-force	For authentication and encryption, a new lightweight protocol is proposed for CAN bus.
CAN Bus	Proposed scheme has high compatibility with existing architectures and testbed experiment is performed	Symmetric Key, HMAC, Trusted Group [97]	No comparative analysis is provided for other types of attacks	Injection, Sniffing, Spoofing	A practical framework is proposed for solving issue of message authentication.
CAN Bus	The proposed scheme has built in fault detection mechanism	CRC, Lightweight Stream Cipher [98]	No comparative analysis is provided for other types of attacks	Masquerade	Proposed scheme utilizes CRC for finding bit errors if any. CAN Data frame part is encrypted using light weight stream cipher.
CAN Bus	The proposed security scheme provide secure environment for CAN-FD and performance is evaluated with microcontrollers and oftware	SHA-256, AES-128, HMAC [99]	No comparative analysis is provided for other types of attacks	Spoofing, Sniffing, Replay	A security architecture is proposed for developing secure communication environment for CAN-FD.
CAN Bus	Backward Compatibility, no need to modify existing nodes	Counter, MAC, 128-bit key [100]	No comparative analysis is provided for other types of attacks	Spoofing, Replay, Injection	An authentication protocol is proposed in which ECUs are allowed to authenticate each other.
CAN Bus	Message authentication for CAN bus with the presence of existing constraints	SHA1, HMAC [101]	No comparative analysis is provided for other types of attacks	Replay, Denial of Service	In the proposed scheme, time stamp as well as HMAC are used for the message authentication.
CAN Bus	The proposed scheme can change the encrypted messages frequently	Symmetric Key (Dynamically Managed) [102]	No comparative analysis is provided for other types of attacks	Replay	In the proposed scheme, payload data is encrypted using symmetric key. Key generators are used to dynamically changing the symmetric key.
CAN Bus	The proposed security framework is hardware-based	PUFs, ECDH [103]	No comparative analysis is provided for other types of attacks	Spoofing, Eavesdropping	In the proposed scheme, ECDH is utilized. In this scheme shared key is not stored.
CAN Bus	The proposed security model block the compromised data on the receiver as well as sender side simultaneously.	Blacklisting, MAC, Whitelisting [104]	No comparative analysis is provided for other types of attacks	Denial of Service, Man in the Middle	A hardware-based security framework is designed. For secure booting, trusted hardware modules are used.
CAN Bus	The proposed scheme is evaluated on several embedded systems environment	128-bit key, ChaskeyMAC, Pre-shared [105]	No comparative analysis is provided for other types of attacks	Spoofing, Replay	An authentication protocol is proposed for CAN-FD, utilizing ChaskeyMAC.
CAN Bus	In the proposed scheme CAN ID is shuffled using NAS frequently.	HMAC, AES-128, SHA-256, Shuffling-CAN ID, AKEP-2 [106]	No comparative analysis is provided for other types of attacks	Replay, Impersonation	In the proposed scheme, attack surface is dynamically shuffled using one time Id.
CAN Bus	Communication security is provided by group-based approach, effective group key management	Keys (Public andPrivate), Gateway-ECU [107]	No comparative analysis is provided for other types of attacks	Spoofing, Sniffing, Replay	A new security architecture is proposed in which Gateway-ECU is used for communication among ECUs.
CAN Bus	Experiment is conducted on real hardware.	GHASH, AES-128 [108]	Delay Issue, no comparative analysis is provided for other types of attacks	Sniffing, Replay, Spoofing	In the proposed scheme, encrypted CAN frames are assigned several different priorities for handling the increased delay in the system.
CAN Bus	The proposed scheme does not require any changes to existing hardware.	AKEP2, MAC, Session Keys [109]	No comparative analysis is provided for other types of attacks	Denial of Service, Masquerade, Bus-Off	A new authentication protocol is proposed. No need of any hardware modifications.
CAN Bus	Design optimization is performed in the proposed scheme for ensuring time critical execution of applications	HMAC 64 bits, Key distribution process based on Diffie-Hellman [110]	No comparative analysis is rovided for other types of attacks	Denial of Service, Injection, Replay, Impersonation, Bus off	The proposed scheme uses HMAC for ensuring security on CAN bus.
CAN Bus	Sender nodes are authenticated using software-based mechanism	AES-128, MAC [111]	No comparative analysis is provided for other types of attacks	Concatenation, Injection, Replay	The proposed scheme utilizes the ordered CMAC buffer for authenticating the CAN frames ID.
CAN Bus	The proposed scheme has two significant contributions, namely sender authentication and effective key management	MAC, Session Keys [112]	No comparative analysis is provided for other types of attacks	Replay, Impersonation	The proposed scheme is characterized with two features, namely authentication of the sender as well as management of keys.
CAN Bus	Overhead of CAN communication is reduced significantly by mixing diversified authentication tags.	SHA-256, MAC, Symmetric Key Cryptography, Bloom Filters [113]	No provision of key distribution and no comparative analysis is provided for other types of attacks	Replay, Man in the Middle	In the proposed scheme, CAN bus data authentication is carried out with the help of Bloom Filters attributes.
CAN, Automotive Ethernet	Mutual identity authentication is provided to all communication parties and session key confidentiality is effectively managed	Symmetric Cryptography, Session Keys, AEAD Algorithm [114]	No comparative analysis is provided for other types of attacks	Eavesdropping, Replay, Man in the Middle, Masquerade	The proposed scheme is featured with efficient authentication as well as secure communication. Session keys are updated regularly.
